# Solution Structure of a Repeated Unit of the ABA-1 Nematode Polyprotein Allergen of *Ascaris* Reveals a Novel Fold and Two Discrete Lipid-Binding Sites

**DOI:** 10.1371/journal.pntd.0001040

**Published:** 2011-04-19

**Authors:** Nicola A. G. Meenan, Graeme Ball, Krystyna Bromek, Dušan Uhrín, Alan Cooper, Malcolm W. Kennedy, Brian O. Smith

**Affiliations:** 1 School of Chemistry, University of Glasgow, Glasgow, Scotland, United Kingdom; 2 Edinburgh Biomolecular NMR Unit, School of Chemistry, University of Edinburgh, Edinburgh, Scotland, United Kingdom; 3 Institute of Molecular, Cell and Systems Biology, University of Glasgow, Glasgow, Scotland, United Kingdom; 4 Institute of Infection, Immunity and Inflammation, University of Glasgow, Glasgow, Scotland, United Kingdom; Institut Pasteur, France

## Abstract

**Background:**

Nematode polyprotein allergens (NPAs) are an unusual class of lipid-binding proteins found only in nematodes. They are synthesized as large, tandemly repetitive polyproteins that are post-translationally cleaved into multiple copies of small lipid binding proteins with virtually identical fatty acid and retinol (Vitamin A)-binding characteristics. They are probably central to transport and distribution of small hydrophobic compounds between the tissues of nematodes, and may play key roles in nutrient scavenging, immunomodulation, and IgE antibody-based responses in infection. In some species the repeating units are diverse in amino acid sequence, but, in ascarid and filarial nematodes, many of the units are identical or near-identical. ABA-1A is the most common repeating unit of the NPA of *Ascaris suum*, and is closely similar to that of *Ascaris lumbricoides*, the large intestinal roundworm of humans. Immune responses to NPAs have been associated with naturally-acquired resistance to infection in humans, and the immune repertoire to them is under strict genetic control.

**Methodology/Principal Findings:**

The solution structure of ABA-1A was determined by protein nuclear magnetic resonance spectroscopy. The protein adopts a novel seven-helical fold comprising a long central helix that participates in two hollow four-helical bundles on either side. Discrete hydrophobic ligand-binding pockets are found in the N-terminal and C-terminal bundles, and the amino acid sidechains affected by ligand (fatty acid) binding were identified. Recombinant ABA-1A contains tightly-bound ligand(s) of bacterial culture origin in one of its binding sites.

**Conclusions/Significance:**

This is the first mature, post-translationally processed, unit of a naturally-occurring tandemly-repetitive polyprotein to be structurally characterized from any source, and it belongs to a new structural class. NPAs have no counterparts in vertebrates, so represent potential targets for drug or immunological intervention. The nature of the (as yet) unidentified bacterial ligand(s) may be pertinent to this, as will our characterization of the unusual binding sites.

## Author Summary

Parasitic nematode worms cause serious health problems in humans and other animals. They can induce allergic-type immune responses, which can be harmful but may at the same time protect against the infections. Allergens are proteins that trigger allergic reactions and these parasites produce a type that is confined to nematodes, the nematode polyprotein allergens (NPAs). These are synthesized as large precursor proteins comprising repeating units of similar amino acid sequence that are subsequently cleaved into multiple copies of the allergen protein. NPAs bind small lipids such as fatty acids and retinol (Vitamin A) and probably transport these sensitive and insoluble compounds between the tissues of the worms. Nematodes cannot synthesize these lipids, so NPAs may also be crucial for extracting nutrients from their hosts. They may also be involved in altering immune responses by controlling the lipids by which the immune and inflammatory cells communicate. We describe the molecular structure of one unit of an NPA, the well-known ABA-1 allergen of *Ascaris*, and find its structure to be of a type not previously found for lipid-binding proteins, and we describe the unusual sites where lipids bind within this structure.

## Introduction

Tandemly repetitive polyproteins (TRPs) are rare in nature. They are produced as large precursor polypeptides, comprising repeated units of similar or identical amino acid sequence that are post-translationally cleaved into a dozen or so copies of functionally similar proteins. Unlike in viral or some neuropeptide and hormone polyproteins, units of TRPs appear to be structurally similar and exhibit similar biochemical activities. The best characterised examples of TRPs are the filaggrins produced by the keratinocytes [1] and the polyprotein allergens of nematode worms (NPAs) [2]. No three-dimensional protein structural information has hitherto been available for a TRP.

Repetitive polyproteins represent an efficient and economical means of synthesizing large quantities of a functional protein. Within a single transcript, multiple copies of the functional protein are encoded, interrupted by regularly-spaced proteinase cleavage sites. This economy of synthesis hypothesis is further supported by the fact that TRPs have very small or no introns in most of the genomic region encoding the tandemly repeated units [1], [2], and the post-translational processing may be similar for both the NPAs and the filaggrins [1], [3], [4], [5], [6]. Since the filaggrins are products of terminally-differentiating apoptotic cells, it has been postulated that the synthesis of proteins as TRPs is an adaptation for cells undergoing deteriorating transcription and translation conditions [1]. But, NPAs are produced in large quantities by organisms in whose tissues there is no sign of programmed cell death [2]. Filaggrins are structural components of keratinocytes and are functionally distinct from NPAs, but it is clear that there is no satisfactory explanation for the adaptive value for the synthesis of proteins as TRPs. Perhaps a more interesting question is why more proteins are not synthesized in this way.

Although NPAs were first identified in human and animal-parasitic nematodes, they are also produced by plant-parasitic and free-living species [7], [8], in which they are synthesized in the intestinal cells and then exported to the pseudocoelomic fluid and the secretions of the worms [2], [8], [9], [10]. In the NPA precursor, post-translational cleavage occurs at regularly-spaced basic motifs (e.g. ^Lys^/_Arg_–Xaa–^Lys^/_Arg_–Arg), similar to the furin cleavage motif at which cleavage is required for activation of filaggrins and some viral proteins [11], [12], [13]. Several species of disease-causing nematodes, including *Ascaris lumbricodes* and *Brugia malayi* of humans and *Dictyocaulus viviparus*, *Ostertagia ostertagi*, *Haemonchus contortus* and *Dirofilaria immitis* of domestic animals [9], [14], [15], [16], [17], [18], [19] express NPAs that are the target of strong immune responses, often of a type associated with hypersensitivities [4], [20], [21]. Examples of the latter range from the acute pulmonary hypersensitivity responses sometimes observed in *Ascaris* infection, to the debilitating chronic elephantiasis pathology of lymphatic filariasis (*B. malayi*). Immune responses to NPAs in infection have been shown to be under strict control of the major histocompatibility complex [22], and there is some evidence that NPAs merit attention for inclusion in vaccines [23]. In the case of humans infected with *A. lumbricoides*, there is epidemiological data that IgE (allergic-type) antibody responses are associated with the development of natural resistance to the infection [24], [25].

All NPA unit proteins examined thus far have significant affinity for small lipids such as fatty acids and retinoids [3], [9], [14], [26], [27]. Such lipids, being relatively insoluble in water and often sensitive to oxidation, require transporter proteins for their safe movement within cells or in extracellular fluids and circulatory systems. An additional importance for parasitic nematodes is that they cannot synthesize complex lipids and must therefore scavenge such nutrients from the host organism, so the NPAs may be crucial for acquisition, protection and distribution of lipids. NPAs also have affinity for pharmacologically active lipids, or their precursors such as arachidonic acid [2], suggesting that sequestration or delivery of these natural signalling molecules may be a mechanism by which parasites can subvert the host immune response.

NPAs are therefore of interest for their relevance to parasitology and to protein science. Using high-resolution protein nuclear magnetic resonance (NMR), we have solved the structure of a single unit of this family of parasite allergens in solution. We demonstrate that ABA-1A, representing the most commonly-repeated unit within the *A. suum* NPA, and a major product of the parasite, adopts a novel seven-helical fold. Further, we identify two discrete hydrophobic cavities within the protein, identified as ligand binding sites with different characteristics.

## Methods

### Expression and purification of recombinant protein

The construct of ABA-1A chosen for NMR studies corresponds to the most C-terminal A-type repeat of the *Ascaris suum* NPA (formally named As-NPA-1A but the more widely used term ABA-1A will be used here), EMBL/GenBank accession number Q06811. Recombinant (r)ABA-1A is extended by five extra amino acids (GSPEF; single letter amino acid code) at the N-terminus of the wild-type sequence. The protein was expressed in *Eschericia coli* in Luria-Bertani broth or Silantes OD2 medium (Silantes GmbH, Munich, Germany) and purified to homogeneity as previously described [28]. Silantes *E. coli* media OD2-CN and OD2-N (Silantes GmbH, Munich) were used to prepare double-labelled (^13^C, ^15^N) and single labelled (^15^N only) samples respectively. The purity of the target protein was estimated to be greater than 98% from both SDS-PAGE electrophoresis and MALDI-TOF and electrospray mass spectrometry. The removal of contaminating ligands from the bacterial expression system was achieved by reverse-phase (RP) chromatography with a C8 stationary phase and water/acetonitrile/trifluoroacetic acid mobile phase, followed by refolding in aqueous buffer.

For the purposes of NMR, unstripped protein was concentrated to approximately 2 mM in 50 mM NaCl, 50 mM sodium phosphate 0.001% (v/v) 50 mM benzamidine, 0.001% (v/v) 50 mM phenylmethylsulfonyl fluoride, pH 7.0. D_2_O was added to a final concentration of 10% (v/v). Approximately 10 µL of 10 mg/mL oleic acid in ethanol was added to each 600 µL NMR sample to ensure saturation of the ligand binding sites. An equivalent volume of ethanol was added to a separate sample of [^15^N]-ABA-1A as a control. Analysis of ^15^N-HSQC spectra confirmed that the chemical shift changes observed upon addition of oleic acid to ABA-1A were due to lipid alone. No evidence of a persistent emulsion was observed; excess fatty acid remained at the sample meniscus and did not adversely affect the quality of NMR data obtained.

For collection of residual dipolar couplings (RDCs), a [^13^C,^15^N]-ABA-1A sample was partially aligned [29] through addition of filamentous phage Pf1 (Profos AG, Regensberg, Germany) at a final phage concentration of 4.8 mg/mL (13.1 Hz ^2^H splitting). The concentrations of protein in the isotropic and aligned samples were 2.5 mM and 1.5 mM respectively.

### NMR data collection and assignment of spectra

All experiments were performed at 308 K using Bruker AVANCE 600 MHz and 800 MHz spectrometers equipped with 5 mm triple-resonance probes and pulsed-field gradients. The WATERGATE tailored selective excitation sequence was typically used for water suppression [30]. The operating temperature of the spectrometer was calibrated to 308 K before each experiment, using a sample of 100% ethylene glycol. Proton chemical shifts were referenced relative to the H_2_O offset frequency and heteronuclear chemical shifts calculated from the proton reference according to the method of Wishart *et al.* [31]. NMR spectra were processed using AZARA (Wayne Boucher, Dept. of Biochemistry, University of Cambridge, http://www.bio.cam.ac.uk/azara) and assigned using ANSIG [32] with subsequent analysis carried out using CCPNmr analysis [33]. Maximum entropy reconstruction [34] was used to enhance resolution of the indirect dimensions of three-dimensional experiments. Assignment was accomplished as previously described [35], ^1^H, ^13^C and ^15^N chemical shift data have been deposited in the BioMagResBank database (http://www.bmrb.wisc.edu) under accession number 6333. ^1^D_NH_, ^1^D_NC′_ and ^2^D_H(N)C′_, ^1^D_C′Cα_, ^1^D_CαHα_ couplings were measured from IPAP-[^15^N]-HSQC [36], HN(α/β-NC′-*J*)-TROSY [37], HN(α/β-COCA-*J*)-TROSY [38] and *J*-(HNCO)CANH [39] respectively. Couplings were evaluated by comparing resonances under isotropic and aligned conditions. Estimates of the average contribution of the dipolar coupling to J_NH_ (and the associated error) were obtained by collecting two independent IPAP-[^15^N]-HSQC datasets.

### Structure calculation

NOE restraints were obtained from 3D ^15^N-NOESY-HSQC and ^13^C-edited ^1^H,^1^H spectra each with100 ms mixing time. Distance restraints were derived from NOESY crosspeaks with the initial mapping from normalised intensity to distance following a 1/r^6^ relationship. NOE distance restraints were incorporated in restrained molecular dynamics calculations using the ambiguous distance restraints formalism [40]. Structures were calculated from randomized initial atomic coordinates using CNS [41] using the PARALLHDG-5.1 force field with PROLSQ non-bonded energy terms [42]. Restraints for the conserved disulfide bond were introduced once the juxtaposition of the cysteine residues was observed in structure calculations based on experimental data. Assignment of prochiral centres was achieved by modification of the protocols to include active prochiral swapping with a Metropolis-style acceptance criterion [43]. Conservative φ, ϕ dihedral angle restraints (−65° ±30°, −40° ±30°) were initially used to define the alpha-helices predicted by CSI [44]. Initial structures were subsequently refined by iteratively filtering the ambiguous distance restraints against the calculated structures to discard duplicate restraints and assignments contributing less than 1%–5% to the total NOE intensity. Residual dipolar coupling (RDC) and hydrogen bond restraints were then introduced. ^1^D_NH_, ^1^D_NC′_, ^1^D_C′Cα_ and ^1^D_CαHα_ restraints were incorporated in the structure calculations using the TENSO module [45] implemented in CNS applying a harmonic potential as previously described [39]. A total of 71 ^1^D_NH_, 70 ^1^D_NC′_, 66 ^1^D_C′Cα_ and 40 ^1^D_CαHα_ couplings were included for residues with {^1^H}^15^N NOE ratios >0.6. Hydrogen bonds restraints were included for amide protons whose signals were still observed in a ^15^N-HSQC spectrum recorded 1 week after redissolution of a lyophilised sample in D_2_O. 25 Hydrogen-bond acceptors were identified by inspection of the NOE-refined structures where supported by NOE data. The average RDC alignment tensor was estimated from this ensemble using PALES [46] and used to incorporate the RDC restraints via the SANI potential [47] in square-well mode. CSI derived backbone dihedral restraints were replaced with restraints produced by DANGLE [48] for the helical regions only. The final ensemble of structures was refined in explicit water after three rounds of NOE disambiguation using ARIA 2.3.

The representative ensemble of structures comprises the 20 lowest energy models from a final round of 100 calculated structures. The quality of these structures was analysed using PROCHECK [49] and their coordinates deposited in the Protein Data Bank (http://www.rcsb.org) under accession code 2XV9.

### ^15^N relaxation measurements

^15^N-relaxation rates, R_1_ and R_2_ were assessed using the method of Kay [50], [51] at field strengths of 600 MHz and 800 MHz. Relaxation delays for assessment of R_1_ were 10.0 ms, 200 ms, 300 ms, 800 ms, 1000 ms and 1300 ms while those for R_2_ were 32.0 ms, 64 ms, 96.1 ms, 160.1 ms, 192.1 ms and 224.0 ms. The first experiment in each series was repeated in order to estimate the inherent error in calculation of crosspeak intensities. Relaxation times T_1_ and T_2_ were calculated using non-linear least squares fitting [52]. Collection of ^15^N-HSQC-heteronuclear NOE experiments with and without saturation allowed extraction of {^1^H}^15^N NOE values. Both saturation and reference experiments were repeated for the purpose of error estimation.

### Spectrofluorimetry

All fluorescence measurements were performed at 25°C in a Perkin-Elmer LS50 or a Spex FluoroMax fluorometer (Spex Industries, Edison, NJ) using 2 mL samples in a silica cuvette. Raman scattering by solvent was corrected where necessary using appropriate blank solutions. The fluorescent fatty acid analogues, 11-(((5-(dimethylamino)-1-napthalenyl)sulfonyl)amino)undecanoic acid (DAUDA) and dansyl-α-aminooctanoic acid (DACA), were obtained from Molecular Probes (c/o Invitrogen, UK) and Sigma (Poole, Dorset, UK), respectively. DAUDA was prepared as a stock solution of approximately 1 mg mL^−1^ in ethanol and freshly diluted in phosphate-buffered saline (PBS; 171 mM NaCl, 3.3 mM KCl, 17 mM Na_2_HPO_4_, 1.8 mM KH_2_PO_4_, pH 7.3) to 1 µM before use, assuming a theoretical extinction coefficient of 4400 M^−1^ cm^−1^. The excitation wavelength (λ_exc_) used was 345 nm.

Titrations were performed as follows: 5–10 µL aliquots of protein at 60–80 µM were added successively to 2 mL DAUDA at 1 µM in PBS and the fluorescence emission spectrum recorded after each addition.. Experiments with DACA followed a similar procedure. Fluorescence data were corrected for dilution and fitted by standard non-linear regression techniques (using Microcal ORIGIN v6.1) to a single non-competitive binding model, as previously described [27], [28].

Protein thermal stability was determined by differential scanning calorimetry (DSC) using a MicroCal VP-DSC (MicroCal Inc./GE Healthcare, Northampton, MA) at a scan rate of 60°C hr^−1^, over a 20–110°C temperature range. Protein solutions, typically ∼1 mg ml^−1^, were dialysed against PBS buffer and degassed briefly before use.

## Results

### Fatty acid binding by recombinant ABA-1A

Recombinant ABA-1A (rABA-1A), expressed and purified as described in the Methods section, has solution properties characteristic of a small, monomeric globular protein similar to the natural material, with sharp and well-resolved NMR spectral features typical of a compact folded protein suitable for high-resolution structure determination.

**Figure 1:**
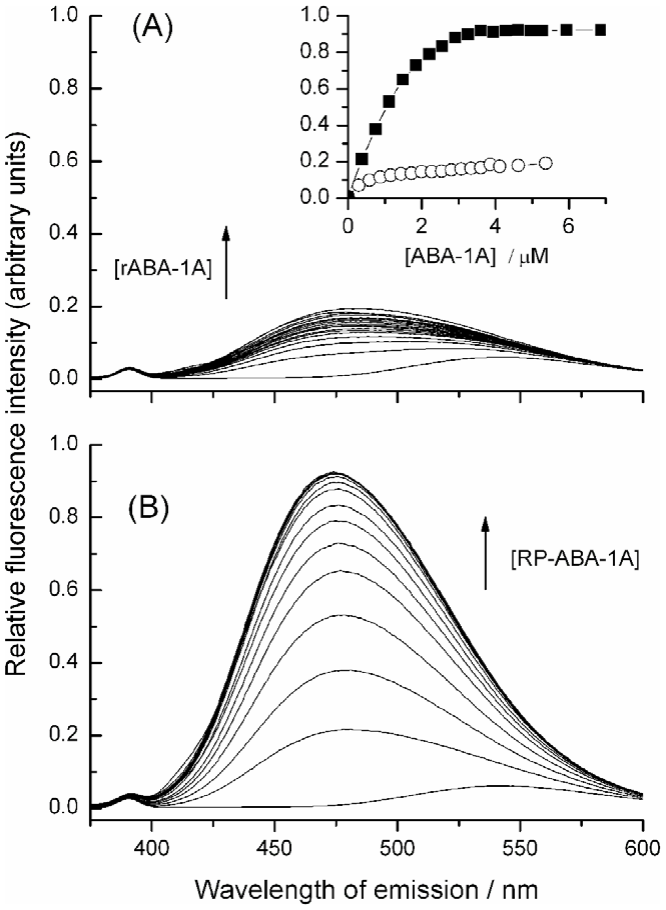
Lipid binding by recombinant ABA-1A before and after removal of resident ligands. Comparison of the fluorescence enhancement of the fatty acid probe, DAUDA, upon binding to recombinant (r)ABA-1A before and after removal of contaminating ligand(s) by reverse phase chromatography. Fluorescence emission spectra of DAUDA (1.39 µM, λ_exc_ = 345 nm) upon additions of increasing concentrations of (A) rABA-1A, and (B) RP-ABA-1A, plotted on the same scale for comparison. The inset shows the increase in DAUDA fluorescence intensity at 475 nm with increasing concentrations of (circles) rABA-1A and (black squares) RP-ABA-1A under identical experimental conditions. Estimated K_D_ for DAUDA binding to RP-ABA-1A is 0.11 (±0.03) µM.

**Figure 2:**
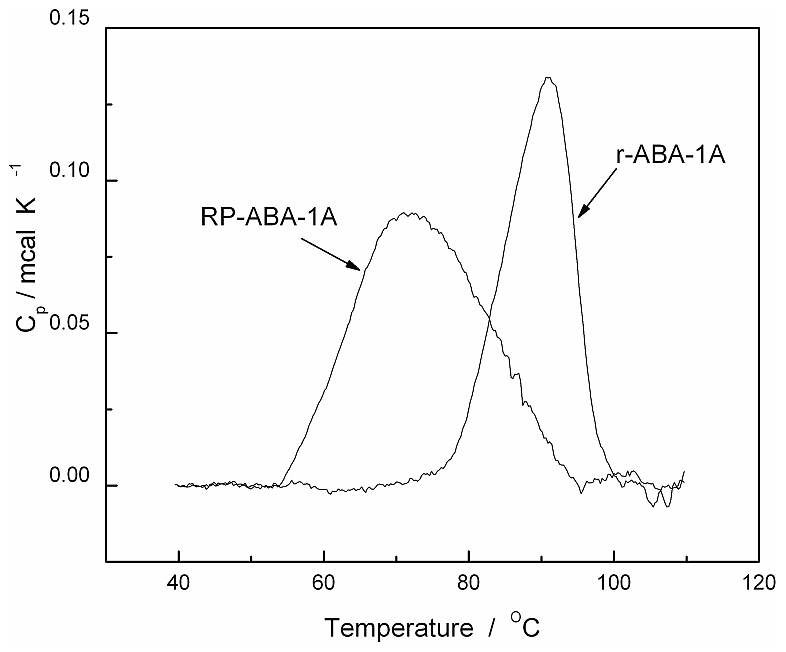
Reduced thermal stability of recombinant ABA-1A upon removal of resident ligand(s). Differential scanning calorimetry (DSC) data showing the higher thermal stability of rABA-1A compared to the same protein (RP-ABA-1A) after treatment to remove endogenous ligands. This is consistent with the presence of bound endogenous ligands stabilizing the native conformation of the recombinant protein.

Prior to more comprehensive NMR studies, the lipid-binding activity of rABA-1A was monitored using DAUDA, a fluorescent fatty acid derivative containing a dansyl fluorophore at the ω-carbon position [27], [53], [54], [55]. In agreement with previous work, on interacting with rABA-1A in solution, DAUDA fluorescence emission underwent a substantial blue-shift (from ∼543 nm to 472 nm; Figure 1), consistent with binding of the fluorophore in a highly apolar protein site [9], [27], [28]. A similar blue-shift (data not shown) was observed using dansyl-DL-α-caprylic acid (DACA) where the fluorophore is adjacent to the carboxylate of the fatty acid, indicating that the entire fatty acid may be internalized and isolated from solvent water [27]. These fluorescence changes can be used to generate experimental binding isotherms from which information about binding affinity and stoichiometry can be derived. As with all previous studies on *Ascaris* and other NPAs [3], [26], [27], fatty acids such as oleic and arachidonic acids displaced DAUDA from the rABA-1A binding site, and the protein also bound retinol (not shown). However, during the initial stages of this work it became clear that the ligand binding properties of recombinant ABA-1A did not precisely match those previously determined for the parasite-derived material (pABA-1A) [27] (Figure 1). In particular, both the apparent fatty acid binding affinity (K_D_) and binding capacity (as indicated by maximal fluorescence intensities) were somewhat lower for rABA-1A than for pABA-1A under similar conditions, with K_D_ for DAUDA binding to rABA-1A typically an order of magnitude weaker than observed with the natural material, for which K_D_ is around 0.1 µM [27], [28]. Moreover, and initially paradoxically, the thermal stability of rABA-1A, as determined by differential scanning calorimetry (DSC) experiments, was significantly higher (T_m_∼90°C) than for pABA-1A, lacking the lower temperature unfolding transition (T_m_∼72°C) seen in the natural material (ref. [27] and Figure 2). This enhanced stability is consistent with a possibility that ligand binding to rABA-1A is inhibited by the presence of an endogenous ligand (or non-natural co-factors), absent from the natural material, but incorporated into the folded protein during the recombinant bacterial expression procedure and retained during subsequent purification stages. We already know that bacterial lipids are bound to other recombinant lipid binding proteins from nematodes [9], [53], [56]. Closer inspection of initial NMR data (see below) revealed the presence of additional, non-protein features consistent with previously unrecognized bound ligand(s). As is known from the thermodynamics of protein interactions (e.g. [54], [57]), ligand binding to native protein will enhance thermal stability without necessarily affecting native conformation. The relatively large increase in T_m_ seen by DSC suggests quite tight binding of these non-natural ligands, as indeed must be the case for them to remain bound throughout the aqueous purification process.

Alternative explanations for these discrepancies may be ruled out as follows. For example, small differences in the N-terminal amino acid sequences of r- versus pABA-1A, introduced as a consequence of the particular expression system (rABA-1A is extended by five amino acids at the N-terminus), might give rise to conformational differences or changes in protein folding stability that could affect ligand binding. However, although such effects might reduce binding affinity, they are unlikely to affect apparent binding stoichiometry. It would also be relatively unusual for these normally-flexible regions at the ends of the polypeptide chain to be directly involved in ligand binding sites. Moreover, our previous work on the ABA-1 of *A. suum* used purified parasite-derived material that would have been heterogeneous in amino acid sequence, albeit comprising NPA units of similar binding characteristics [14], [27], and possibly contaminated with parasite-derived lipids that would have compromised the estimation of dissociation constants. Partial unfolding or misfolding of the recombinant protein could plausibly reduce binding affinities, though this is seemingly ruled out by the quality of the NMR data (see below) that indicate a homogeneous population of well-folded molecules in solution.

Removal of the endogenous ligand(s) was accomplished by reverse-phase chromatography of unfolded protein, followed by careful refolding to yield protein (RP-ABA-1A) with binding and stability properties much closer to natural material (see Figures 1 and 2), separate experiments [58] having established that denatured ABA-1A refolds rapidly and efficiently when returned to non-denaturing conditions. In particular, removal of endogenous ligand(s) resulted in significantly higher fluorescence emission of DAUDA at saturating protein concentrations, with binding affinity (K_D_≈0.1 µM) comparable to natural parasite-derived ABA (Figure 1). RP-ABA-1A also has a much lower thermal stability (Figure 2). This is consistent with the hypothesis, supported in more detail by NMR data (see below), that stripping of endogenous ligands from recombinant rABA-1A leads to higher binding capacity and increased binding affinity by exposure of a second, higher affinity fatty acid binding site.

### The solution structure of ABA-1A reveals a novel fold

**Figure 3:**
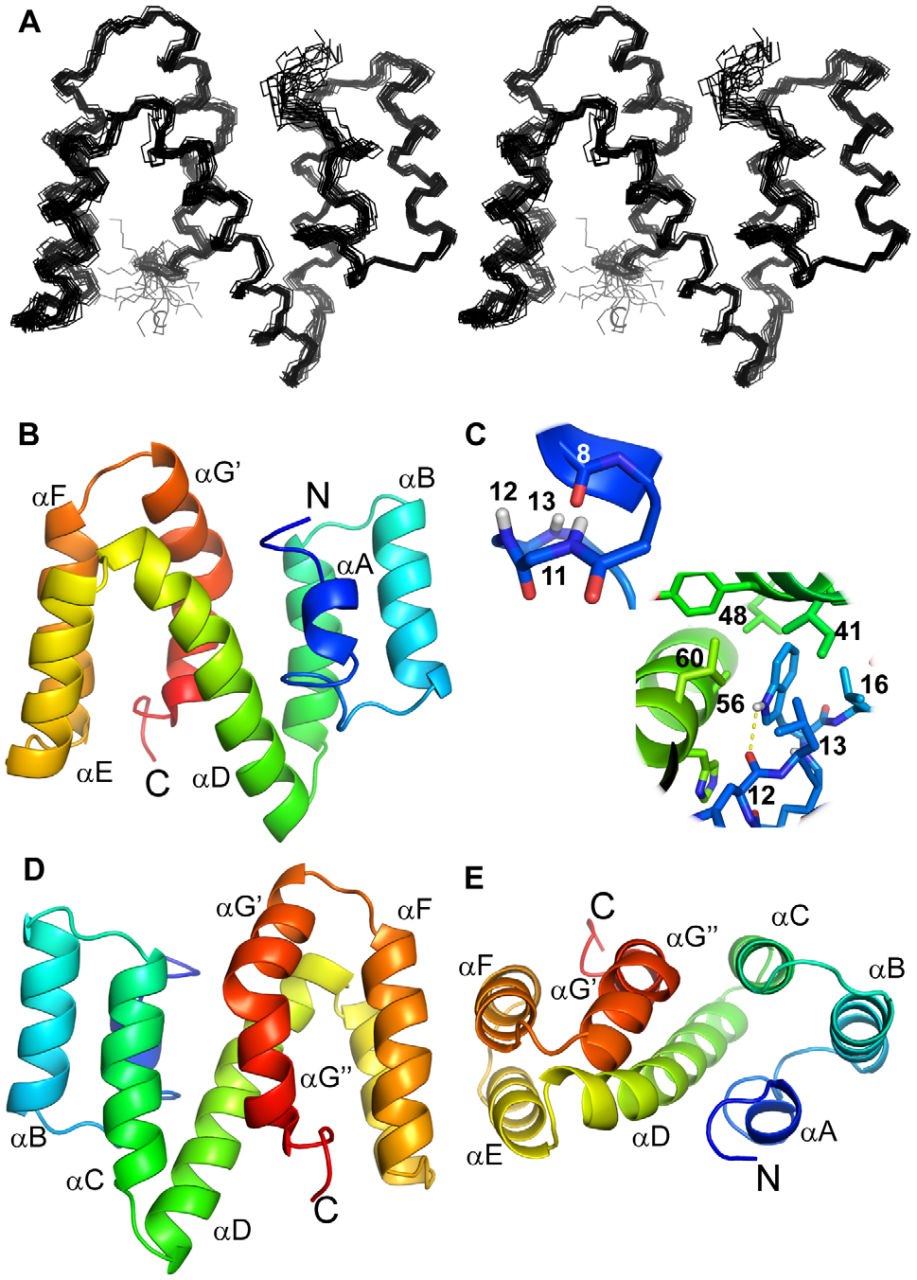
The nuclear magnetic resonance structure of ABA-1A in solution. (A) A stereo plot (wall-eyed) depicting the 20 lowest energy structures (from a total of 100) superimposed on the backbone heavy atoms of residues 4–126. (B, C, D) Three cartoon views (A and E rotated 180° about the y-axis and 90° about the x-axis with respect to (B) of the representative structure of ABA-1A with a color progression from blue (N-terminus) to red (C-terminus). (C) Enlargements of the wide helical turn at the end of α-helix A (upper inset) and the local environment of the absolutely conserved position Trp15 of NPAs (lower inset). Figures prepared using PyMol.

The structure of ABA-1A was solved by NMR spectroscopy. A total of 4242 NOE-derived distance restraints were used to calculate the structure of ABA-1A, of which 2069 were unambiguous or manually assigned, and 2173 ambiguous restraints in the final refinement (Table 1). These were supplemented during later rounds of structure calculation by restraints derived from 247 RDCs (71 ^1^D_NH_, 70 ^1^D_NC′_, 66 ^1^D_C′Cα_ and 40 ^1^D_CαHα_) observed in a sample partial aligned in Pf1 filamentous bacteriophage [59] and 25 inferred hydrogen-bonds restraining slowly exchanging amide protons to hydrogen bond acceptors identified from preliminary structures. The final ensemble of structures (Figure 3A) comprises the 20 lowest in energy, ranked on the basis of the experimental restraint energy term, of 100 structures calculated. The structures were refined in explicit water [60] and superposed and the structure closest to the mean of the ensemble was chosen as a representative.

**Table 1:** Structural statistics for the 20 lowest energy structures.

	Ensemble	Closest-to-mean
Total NOE restraints	4242	
Ambiguous	2173	
Unambiguous/assignable	2069	
Intra-residue	1046	
Inter-residue	1013	
Sequential (|*i–j*| = 1)	457	
Non-sequential (|*i*–*j*|>1)	566	
Total RDC restraints	247	
^1^D_NH_	71	
^1^D_NC′_	70	
^1^D_C′Cα_	66	
^1^D_CαHα_	40	
Hydrogen bonds	25	
Coordinate r.m.s.d (Å)^b^		
Backbone heavy atoms^a^	0.575	0.388
All heavy atoms^a^	0.963	0.817
Parameter r.m.s.d from idealised geometry (mean and s.d.)		
Bond lengths (Å)	0.00435±8.8×10^−5^	0.0043
Bond angles (°)	0.663±0.015	0.642
Impropers (°)	2.18±0.078	2.07
Dihedrals (°)		
Violations (mean and s.d.)		
Distance restraint violation (Å)	0.033±0.007	0.031
RDC Q factor	0.122±0.005	0.115
Ramachandran statistics (%)		
Most favoured	90.1	93.4
Additionally allowed	9.2	5.7
Generously allowed	0.4	0.0
Disallowed	0.3	0.8

a Residues 4–126.

b To the unbiased mean structure.

The protein forms a compact, globular fold consisting of a long central helix (D) that participates in two flanking helical bundles (ABCD) and (DEFG) (Figure 3B, D, E). All helices are connected in an up-down fashion with inter-helical angles exceeding 140° [61] by very short linkers that typically incorporate a glycine residue. The helices are very regular (see Tables S1 and S2 for interhelical angles and regularity parameters) with the exception of helix G, which is interrupted by a 60° kink at Gly117. Each bundle is maintained by a hydrophobic core involving the central helix. Core I (Leu13, Trp15, Leu16, Ile41, Leu42, Tyr44, Ala56, His59, Leu60, Ile123) centres on the conserved NPA tryptophan, Trp15 and is supplemented by residues from both helices ABC and helix D. Core II (Ile67, Leu68, Val71, Val72, Ala77, Leu80, Ala100, Leu101, Tyr112, Ile113, Ala114, Phe116) involves interactions between the central helix and the C-terminal helix bundle and tethers the lipid-binding cleft to the main body of the structure. The residues at these positions are generally conserved as hydrophobic between NPA units of different species of parasitic nematode, and also between the N- and C-terminal regions of ABA-1 (refs. [6], [27] and also below).

RDC restraints were essential to define the orientation of helices F and G, relative to the rest of the structure. There are few long range NOE restraints involving this stretch of sequence and the conformation of these elements is poorly defined in structures calculated from NOEs alone.

^15^N backbone dynamics show that there are few regions of the protein that are intrinsically flexible, as shown by the flat profile of R_1_ and R_2_^15^N relaxation rates and ^1^H-^15^N NOE data (Figure S1). Indeed, only the terminal regions of the protein and a few residues either in loop regions, or otherwise exposed, deviate from average values, and these do so in a way that would be consistent with motion on the picosecond to nanosecond timescale. Outside the termini and loops, Lys81 shows a small but significant dip in heteronuclear NOE ratio and R_2_. Inspection of the structure shows that this residue lies on the outer face of helix E where it is exposed to possible exchange with solvent water.

The observed pattern of helices differs from other helical lipid-binding proteins of known structure, such as ns-LTPs, ACBP and serum albumin [52], [62], [63]. A comparison of the ABA-1A model with proteins of known structure using distance matrix alignment (DALI) [64] failed to yield any significant hits. A few of poor (RMSD>3.5 Å) candidate matches from the protein structure databank (PDB) were selected by the program. Closer inspection showed these matches were based on the orientational similarity between helical fragments of both protein structures and not any overall structural similarity to ABA-1A. It therefore appears that the ABA-1A fold has not previously been observed and that NPAs belong to a structurally novel class of lipid-binding protein.

### The ABA-1A structure reveals two ligand binding cavities with distinct characteristics

**Figure 4:**
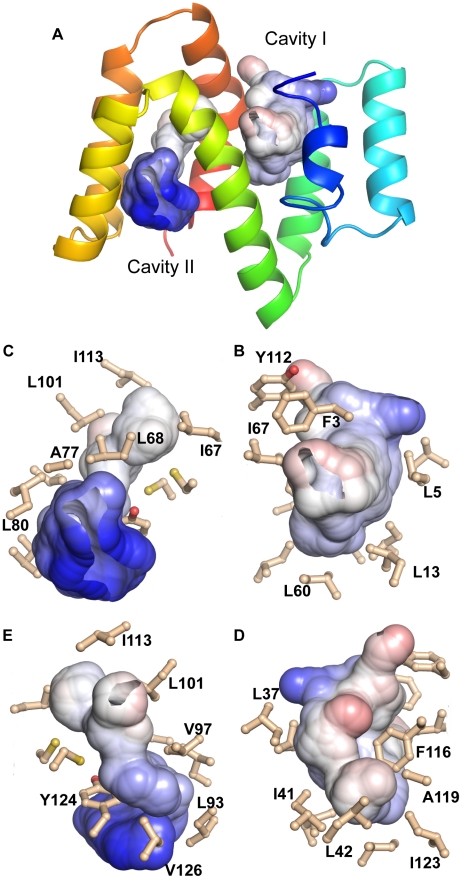
ABA-1A's ligand binding cavities. Molecular surfaces of the atoms lining the ligand binding cavities (probe radius 1.4 Å, cavities truncated at 3 probe radii) coloured by contact potential (blue, positive, graded through white, apolar, to red, negative). (A) Ribbon representation of ABA-1A with the binding cavities added, oriented with the N-terminal domain to the right as in Figure 3B. (B and C) The cavities in the same orientation but enlarged and with the residues lining the cavities displayed as sticks, illustrating the dominant apolarity of the cavities and the cluster of positively charged sidechains at the entrance of Cavity II. (D and E). The cavities rotated 180° about the vertical axis. Note that Trp15 does not contribute to any cavity surface.

**Figure 5:**
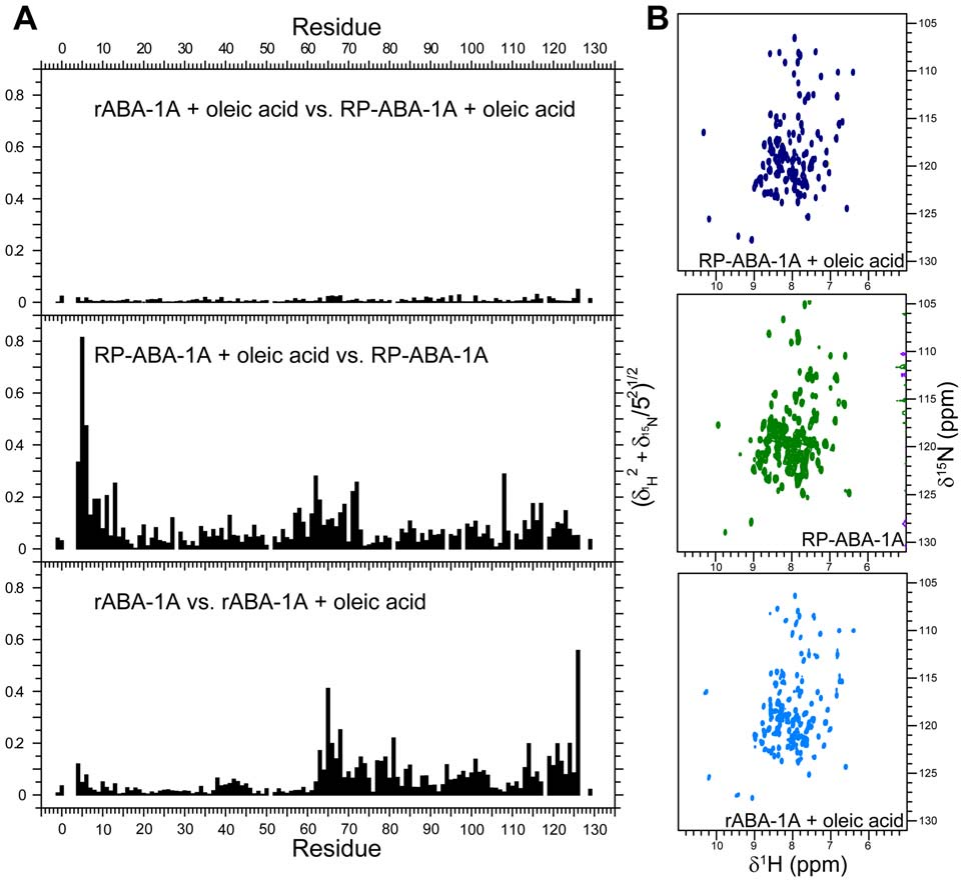
NMR evidence for ligand binding by rABA-1A. A) Backbone amide chemical shift perturbation (√[|δ^1^H|^2^+(0.2|δ^15^N|)^2^]) reporting on changes in structure between different forms of ABA-1A: recombinant ABA-1A purified from *E. coli* (rABA-1A); rABA-1A saturated with oleic acid; rABA-1A stripped of resident ligand(s) by reverse phase chromatography (RP-ABA-1A); and RP-ABA-1A saturated with oleic acid. Saturation of either rABA-1A or RP-ABA-1A with oleic acid gives rise to essentially identical backbone amide chemical shifts and therefore structures. Compared with the oleic acid bound form, rABA-1A has different shifts in its C-terminal half indicating that cavity II is not occupied, or occupied by a different ligand in this form. In contrast RP-ABA-1A displays shifts in both N-and C-termini implying that both cavites are empty in this form. B) ^15^N HSQC spectra of RP-ABA-1A, rABA-1A and RP-ABA-1A saturated with oleic acid.

Unexpectedly, the three dimensional structure of ABA-1A contains two significant cavities, cavity I enclosed by helices ABC&D and cavity II by helices DEF&G (Figure 4). Both cavities are of similar size with approximately 490 Å^3^ accessible to a 1.925 Å probe (equivalent to a CH_2_ group) in both, extending to 805 Å^3^ and 880 Å^3^, respectively, accessible to a 1.4 Å probe (equivalent to a water molecule) [65]. Cavity I is mainly buried within the protein with the more accessible volume lined by the sidechains of largely hydrophobic residues (F3, L5, L13, L37, I41, G63, I67, Y112, F116). The more inaccessible volume behind F116 is lined by L42, L60, A119 & I123. The entrance to the cavity is surrounded by more hydrophilic residues, with a single formal positively charged residue, K34 at the primary opening opposite the most inaccessible volume and H12 at the secondary opening between helices A and D. Cavity II is more open to the solvent and is also lined by sidechains of hydrophobic residues (G62, L68, A77, L80, L93, V97, Y124 & V126) as well as the non-polar parts of K61, R65, K81, and K84. Again there is a less accessible, more hydrophobic volume behind the Y124 aromatic ring lined by C64, I67, L101, I113, G117 and C120. In both cases, there is evidence that the deep parts of the cavities are occupied when oleic acid is added (Figure 5). The solvent-proximal edges of cavity II are decorated with lysines and the single arginine residue. A hydrophobic ligand such as a fatty acid will therefore be oriented in either of the cavities with its hydrophobic region embedded within the cavity, and its charged headgroup (e.g a carboxylate or hydroxyl) anchored at, but opposite to that found in most cytoplasmic fatty acid binding proteins [52], [66].

Thus, ABA-1A contains two potential binding sites for the hydrophobic ligands that it is known to bind. Oleic acid, with which the NMR samples were saturated, has a molecular volume of approximately 300 Å^3^ [67], implying that each cavity is capable of accommodating at least one such molecule. Further evidence for independent ligand binding by each of the cavities comes from a number of sources. The chemical shifts of NMR resonances of residues in both the N- and the C-terminal halves of recombinant ABA-1A that has been fully stripped of resident ligands by reverse phase HPLC are perturbed upon saturation of the protein with oleic acid (Figure 5). Addition of DAUDA principally perturbs resonances in the C-terminal half [58] in a similar manner to that observed when oleic acid is added to unstripped ABA-1 (Figure 5). Also, NOE crosspeaks are observed between the sidechains of residues lining cavity I in unstripped protein, and NMR resonances that cannot be assigned to the protein itself (not shown); these unassigned resonances lie at around 5.2 ppm, a typical shift for vynilic protons (see Figure S2). Cavity I is probably therefore occupied by an as yet unidentified hydrophobic entity that binds with high affinity, originating from either the synthesising bacterium or the culture medium. It is clear, however, that the two cavities differ in their ligand binding propensities such that cavity II binds fatty acids and the bulkier DAUDA (this study and ref. [68]), whereas cavity I binds an unknown type of ligand or ligands at high affinity, though it can also bind less bulky oleic acid when the other ligands are removed. It would be interesting to examine chemical shifts in stripped protein saturated with retinol, as an example of another class of ligands that ABA-1A is known to bind. It is important to realise, however, that a true understanding of the binding sites of this and other NPAs aimed at, for instance, drug development, will require the use of procedures to completely remove ligands derived during production of the recombinant protein.

## Discussion

We describe the solution structure of ABA-1A, a unit of the nematode polyprotein allergen (NPA) of *A. suum*, the amino acid sequence of which is almost identical to that of the large roundworm of humans, *A. lumbricoides*. We find that the protein exhibits a novel fold, we localise the likely binding sites for fatty acids and retinol (Vitamin A), and identify the amino acid sidechains probably involved in interacting with the small lipids that the NPAs may transport within parasitic nematodes, or in their interaction with host tissues. Unexpectedly, we find that the protein possesses two discrete binding sites that probably bind different, though overlapping, sets of hydrophobic ligands. NPAs are only found in nematodes, and this is the first unit of any tandemly repetitive polyprotein yet reported.

### Origin of NPA units

**Figure 6:**
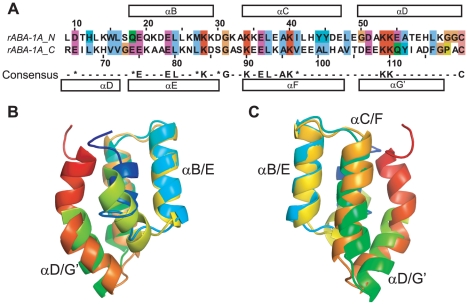
ABA-1A structure and sequence support ancestral gene duplication. A) Structure-based sequence alignment of N- and C-terminal halves of ABA-1A. B) and C) 180° rotated views of a superposition of the N- and C-terminal halves of ABA-1A.

The overall structure of ABA-1A is a flattened, compact, roughly disc-shaped molecule, comprising seven α-helices and no β structure. Sequence analysis has indicated that NPA units such as ABA-1A themselves arose from an ancient duplication event [6], [27], [69]. We now see that this sequence duplication is also reflected at the level of the molecular structure (Figure 6); the putative internal duplication point within ABA-1A occurs precisely halfway along the central helix D, and the amino acids occurring at turns between helices in the N-terminal half coincide in sequence alignments [6], [27], [69] precisely with amino acids at corresponding turns between the C-terminal helices. Moreover, the arrangement of the helices in each half of the protein is identical, with the two halves of the protein inverted, as would be expected if the C-terminus of one were fused to the N-terminus of another at the midpoint of helix D. Thus, the N- and C-termini of the complete protein are related by two-fold rotational pseudosymmetry about an axis perpendicular to the centre of helix D. Each half of the protein forms a small four helix bundle, each with a cavity opening to the exterior, the cavity/pocket in the C-terminal half being larger and more open to the exterior than that of the N-terminal half. This close structural similarity between the two halves is demonstrated graphically by superposition of the two structures (Figure 6). This, together with other sequence-based considerations, reinforces the idea that NPA units arose from a duplication [6], [69].

### ABA-1A and other small lipid-binding proteins

The NPAs present a new type of fold for transporting small lipids, and were the first lipid-binding proteins found to be synthesized as polyprotein precursors. The best understood lipid binding/transporter proteins of the size of NPA units are those of the FABP/P2/CRBP/CRABP family of β-barrel proteins that are intracellular transporters of small lipids, and which seemingly occur throughout the metazoan phyla [70]. Curiously, nematodes so far present the only examples of members of this family of proteins that are extracellular [71], [72]. Nematodes also exhibit a family of larger helix-rich lipid transporter proteins, the FAR proteins, of which there are eight distinct sub-types [53], [56], [73]. The structure of one of these has recently become available [68]. The FARs, like NPAs, bind fatty acids and retinol. Soluble lipid binding proteins that are as helix-rich as NPAs include the serum albumins of vertebrates and the small lipid binding and storage proteins of plants. Albumin is a much larger protein, ∼67 kDa, and is the predominant transporter of fatty acids in mammalian blood, although it has a broad range of other binding properties. Like NPAs, serum albumin is also thought to have arisen from duplication events [52]. The small (∼7 kDa) lipid transporter proteins of plants are also helix-rich and have important roles in lipid storage and transport within plants, in addition to transmitting signals between distant tissues in plants [74], [75]. These also show no signs of any phylogenetic relationship with NPAs, although their size is commensurate with the small ancestral protein postulated to have given rise to an NPA unit by duplication [6].

### Interpretation of mutagenesis results and residue conservation

This newly-derived structure for ABA-1A now helps explain some of the previous observations on biophysical properties and the effects of site-directed mutagenesis on NPAs. For example, the absolutely conserved single tryptophan residue (Trp15) in all NPA units (see Figure S3) exhibits an intrinsic fluorescence emission wavelength that is extremely blue shifted with respect to the solvated residue, and analysis using quenching agents indicates that this Trp is excluded from contact with solvent in the folded protein [27], [76]. Tryptophan fluorescence in proteins is strongly influenced by factors including local peptide sequence, conformation, and polarity of the surrounding environment [77], [78]. The remarkable blue shift in ABA-1A places it at the extreme edge of the range observed in proteins, in a class identified [78] as those for which the Trp sidechain is totally buried, with no exposure to water. This is confirmed in the NMR structure, which shows that Trp15 sits at a hairpin bend of the N-domain with the indole sidechain firmly held in the interior of the protein, surrounded by apolar side chains, and hydrogen bonded via the indole nitrogen to a backbone carbonyl (Figure 3C). The latter interaction may add further to the blue shift by dipole-dipole interaction with the Trp transition dipole. This deep burial, and the absolute conservation of this position in NPAs, highlights the potentially significant structural role played by Trp15. It is therefore perhaps surprising that we see no evidence of involvement of Trp15 in any supposed function of the protein. Although it has been reported that ligand binding alters the Trp fluorescence emission in another NPA [76], this has not been consistently found for ABA-1A, and the position of Trp15 in the structure is quite remote from the ligand binding site(s). Furthermore, replacement of Trp15 with Arg does not affect the protein's binding activity for DAUDA and other ligands tested [28]. Thus, the evolutionary conservation and structural location of Trp15 suggests that its role might relate to other processes, such as a role in the folding and stability of the proteins. For example, as suggested in other rapidly folding protein systems [79], this Trp and associated hydrophobic residues might act as a nucleus for folding of the polypeptide chain as it emerges from the ribosome. This might be particularly important in the polyprotein context to prevent aggregation or entanglements with other units of the chain. Our direct observations on the rate of folding of ABA-1A do indeed indicate extremely rapid re-folding of ABA-1A following unfolding in GdnHCl [58].

The new structure explains some of the effects of previously reported genetic and chemical manipulations of ABA-1A [28]. Mutation of the highly conserved Trp15, Gln20 and Leu42 (see Figure S3) residues had varied effects. Changes at positions 15 and 20 did not affect ligand binding, but did compromise the resistance of the protein to thermal unfolding and chemical denaturation. Mutation at Leu42, however, not only abolished ligand binding, but also dramatically destabilised the protein. Leu42's sidechain is directed towards, and is a key residue in the interaction between, helices C and G′. The disulphide bond between the two (absolutely conserved) cysteines (see Figures S3 and S4) in NPAs tether helix G′ to the main central helix D. Their central position within the structure is therefore understandably reflected in a substantial destabilisation of rABA-1 and abrogation of ligand binding upon reduction and alkylation of the protein, although they are not directly involved in either binding cavity. Lastly, that the C-terminal cavity in ABA-1 contains a self-standing binding site for the bulky DAUDA is confirmed by previous work showing that when constructs comprising separate N- and C-terminal domains of ABA-1 were analysed for DAUDA binding, only the latter did so, and a recombinant form of the NPA of the filarial nematode *Brugia malayi* in which the first 16 amino acids were absent still bound DAUDA [26], [28].

The NPA units of the filarial nematode of humans, *B. malayi* and *Wuchereria bancroftii*, are N-glycosylated [18], [80], but this is not true for ABA-1 of *Ascaris* [27]. The sites at which the *Brugia* and *Wuchereria* NPAs are glycosylated map to position Glu47 on the structure of ABA-1A, which is an outward facing side chain on helix C, a position at which a large glycosyl group could be positioned without modifying the structure of, or ligand binding by, the protein unit.

### Why tandemly repetitive polyproteins?

The adaptive value in producing proteins as TRPs remains mysterious, and the structure we report for an NPA unit does not provide a solution to the problem. TRPs might have been the ancestral form of proteins that appear to have internal duplications (such as serum albumin of vertebrates), yet NPA arrays are very similar across the nematodes and there is no sign that multidomain proteins have evolved from them (although there is evidence that some units are not separated post-translationally [2], [15]. Ascarid and filarial nematode parasites are unusual in having a simplified array of NPA units that comprise predominately units of identical or closely similar amino acid sequences [4], [6], [9]. This is not true of many other groups of animal and plant-parasitic nematodes, or of free-living species, in which the unit sequences are highly diverse [2], [15].

The fact that the individual units of ABA-1A are flattened in tertiary structure and with very short linkers (typically about four amino acids) might suggest that the units, when still in the polyprotein form following extrusion from the ribosome, stack or associate face-to-face following initial folding, such that their binding sites are not exposed for loading until proteolytic separation occurs in a subsequent cell compartment. There are, however, signs neither of shape or charge complementarity, nor surface charge bias, or large non-polar surface patches that would suggest that adjacent units in the nascent polyprotein would either attract or repel each other.

To conclude, the tandemly repeated unit of the *Ascaris* ABA-1 NPA is shown here to comprise a lipid binding protein of novel structure, and that it contains two binding sites that differ in character, although both probably transport hydrophobic or amphipathic ligands. One of these binding sites is populated with a ligand that binds at high affinity in the recombinant protein, possibly derived from the bacterial cultures. Knowing more about what this high affinity ligand(s) is in NPAs could be useful in designing drugs against parasitic nematodes because the N-terminal binding site appears to be unusual and perhaps more specialised than is the C-terminal binding site. The NPAs are renowned for their immunodominance and propensity to be allergens in the context of nematode infections, and, while there are as yet no consensus structural features that makes a given protein type more likely or not to be allergenic, this new structure might contribute to the elucidation of such a consensus that might be different for infection-associated than for environmental allergens.

## Supporting Information

Table S1 Interhelical angles (in degrees) calculated using QHELIX ^[1]^
(0.04 MB DOC)

Table S2 Helix quality calculated using QHELIX ^[1]^
(0.03 MB DOC)

Table S3 The Nematode Polyprotein Allergen (NPA) units used to create the multiple alignments in Figures S3 and S4.
(0.03 MB DOC)

Figure S1 The boxes in the upper panel represent the extent of the α-helices. The flat NOE, R_1_ and R_2_ profiles for the structured regions of the protein indicate that the ligand bound form of the protein is relatively rigid on the ps-ns timescales.
(0.95 MB EPS)

Figure S2 Intermolecular NOEs between ABA-1A residues lining cavity I and an oleic acid vinylic resonance at ∼5.25 ppm. A selected strips from the ^13^C 3D NOESY spectrum for the indicated NMR resonances (blue: positive contours; orange: negative contours) that exhibit NOE crosspeaks to a resonance at ∼5.25 ppm. The dashed red line indicates the ^1^H chemical shift of the vynilic resonance that can be identified from its natural abundance signal in the ^13^C-HSQC spectra of the complex.
(2.28 MB EPS)

Figure S3 Multiple alignment of the N-terminal halves of NPA units. The sequences are from thirteen species of animal and human parasites, one plant parasite and one free-living species. The sequences of these units are highly divergent and alignments are more informative if created with the N-terminal halves (this Figure; unit names with suffix ‘_N’), ending immediately after the first Cys in the sequences, and C-terminal halves (Figure S4; unit names with suffix ‘_C’) treated separately - see Figure 6 of the main paper for the structural indications that modern day NPA units derive from an ancient duplication event as originally postulated from the ABA-1A sequence [1]. All of the units in the NPA of the cattle parasite *Dictyocaulus viviparus* and the free-living *Caenorhabditis elegans* are included, both of which comprise units with highly divergent amino acid sequences. Only partial information on the arrays is available for most parasite species, and sequences of one unit from each is included, except for the two divergent units known from *Ascaris suum*, in which the units are otherwise almost identical [2]. The units are labeled according to the standard nomenclature for nematode genes and proteins, such that ABA-1A is here labeled As-NPA-1A. The alignment emphasizes the complete conservation of the position of Trp15 in the ABA-1A structure (and position 25 in the alignment), including the adjacent Leu or Met, even in the unusual truncated Dv-NPA-1H unit of *D. viviparus*. The two cysteines are also absolutely conserved (this Figure and Figure S4), with, again, the exception of the truncated repeat in *D. viviparous*. The only equivalently conserved position is Gln at position 20 in the structure (and 30 in the alignment), which is replaced just once, with a Glu, a change that can be achieved with a single DNA codon base change. Site-directed substitutions at these positions have various disruptive effects on the thermal stability or ligand binding of ABA-1A [3]. No other positions show similar levels of conservation, although a few others exhibit strong conservation of amino acid type (e.g. position 61 of the N-terminal halves (this Figure), and 11 and 47 of the C-terminal half alignment (Figure S4)). The unusual short unit of *D. viviparus*, Dv-NPA-1H, aligns better with N- rather than the C-terminal half units - it ends in a consensus cleavage site at which the units are separated posttranslationally and then trimmed back [4], that most of the full-length units also exhibit (see Figure S4). Some units have unusual histidine-rich C-terminal extension peptides (Figure S4), for which we have unpublished information that they bind certain divalent metal ions such as zinc. The sequences are named in the alignment and extracted from protein databases as listed below. Those units that have been shown to have ligand binding propensities similar to that of ABA-1A (As-NPA-1A) are As-NPA-1B [2], Dv-NPA-1L [5], Bm-NPA-1 [6], and some of the units from the *C. elegans* array (Ce-NPA-1) (our unpublished data). The alignment was created using MultAlin (http://multalin.toulouse.inra.fr/multalin/multalin.html) set for the default Blosum62 substitution matrix; high consensus indicated in red (upper case in consensus line if complete conservation of that amino acid position), low consensus in blue, neutral, black; in the consensus line $ is anyone of L or M, # is anyone of N, D, Q or E. See Table S3 for a list of the species form which the NPA unit sequences were obtained, the labeling of the units in the figure, and the database accession codes. REFERENCES 1. Spence HJ, Moore J, Brass A, Kennedy MW (1993) A cDNA-Encoding Repeating Units of the ABA-1 Allergen of *Ascaris*. Molecular and Biochemical Parasitology 57: 339–344. 2. Moore J, McDermott L, Price NC, Kelly SM, Cooper A, et al. (1999) Sequence-divergent units of the ABA-1 polyprotein array of the nematode *Ascaris suum* have similar fatty-acid- and retinol- binding properties but different binding-site environments. Biochemical Journal 340: 337–343. 3. McDermott L, Moore J, Brass A, Price NC, Kelly SM, et al. (2001) Mutagenic and chemical modification of the ABA-1 allergen of the nematode *Ascaris*: Consequences for structure and lipid binding properties. Biochemistry 40: 9918–9926. 4. Kennedy MW (2000) The nematode polyprotein allergens/antigens. Parasitology Today 16: 373–380. 5. Kennedy MW, Britton C, Price NC, Kelly SM, Cooper A (1995) The DVA-1 Polyprotein of the Parasitic Nematode *Dictyocaulus viviparus* - a Small Helix-Rich Lipid-Binding Protein. Journal of Biological Chemistry 270: 19277–19281. 6. Kennedy MW, Allen JE, Wright AS, McCruden AB, Cooper A (1995) The Gp15/400 Polyprotein Antigen of *Brugia malayi* Binds Fatty- Acids and Retinoids. Molecular and Biochemical Parasitology 71: 41–50.
(0.39 MB TIF)

Figure S4 A multiple alignment of amino acid sequences of C-terminal halves of NPA units. The sequences are from thirteen species of animal and human parasites, one plant parasite and one free-living species. The sequences of these units are highly divergent and alignments are more informative if created with the N-terminal halves (Figure S3; unit names with suffix ‘_N’), ending immediately after the first Cys in the sequences, and C-terminal halves (this Figure; unit names with suffix ‘_C’) treated separately - see Figure 6 of the main paper for the structural indications that modern day NPA units derive from an ancient duplication event as originally postulated from the ABA-1A sequence [1]. All of the units in the NPA of the cattle parasite *Dictyocaulus viviparus* and the free-living *Caenorhabditis elegans* are included, both of which comprise units with highly divergent amino acid sequences. Only partial information on the arrays is available for most parasite species, and sequences of one unit from each is included, except for the two divergent units known from *Ascaris suum*, in which the units are otherwise almost identical [2]. The units are labeled according to the standard nomenclature for nematode genes and proteins, such that ABA-1A is here labeled As-NPA-1A. The alignment emphasizes the complete conservation of the position of Trp15 in the ABA-1A structure (and position 25 in the alignment), including the adjacent Leu or Met, even in the unusual truncated Dv-NPA-1H unit of *D. viviparus*. The two cysteines are also absolutely conserved (this Figure and Figure S3), with, again, the exception of the truncated repeat in *D. viviparous*. The only equivalently conserved position is Gln at position 20 in the structure (and 30 in the alignment), which is replaced just once, with a Glu, a change that can be achieved with a single DNA codon base change. Site-directed substitutions at these positions have various disruptive effects on the thermal stability or ligand binding of ABA-1A [3]. No other positions show similar levels of conservation, although a few others exhibit strong conservation of amino acid type (e.g. position 61 of the N-terminal halves (Figure S3), and 11 and 47 of the C-terminal half alignment (this Figure). The unusual short unit of *D. viviparus*, Dv-NPA-1H, aligns better with N- rather than the C-terminal half units - it ends in a consensus cleavage site at which the units are separated posttranslationally and then trimmed back [4], that most of the full-length units also exhibit (this Figure). Some units have unusual histidine-rich C-terminal extension peptides, for which we have unpublished information that they bind certain divalent metal ions such as zinc. The sequences are named in the alignment and extracted from protein databases as listed below. Those units that have been shown to have ligand binding propensities similar to that of ABA-1A (As-NPA-1A) are As-NPA-1B [2], Dv-NPA-1L [5], Bm-NPA-1 [6], and some of the units from the *C. elegans* array (Ce-NPA-1) (our unpublished data). The alignment was created using MultAlin (http://multalin.toulouse.inra.fr/multalin/multalin.html) set for the default Blosum62 substitution matrix; high consensus indicated in red (upper case in consensus line if complete conservation of that amino acid position), low consensus in blue, neutral, black; in the consensus line $ is anyone of L or M, # is anyone of N, D, Q or E. See Table S3 for a list of the species form which the NPA unit sequences were obtained, the labeling of the units in the figure, and the database accession codes. REFERENCES 1. Spence HJ, Moore J, Brass A, Kennedy MW (1993) A cDNA-Encoding Repeating Units of the ABA-1 Allergen of *Ascaris*. Molecular and Biochemical Parasitology 57: 339–344. 2. Moore J, McDermott L, Price NC, Kelly SM, Cooper A, et al. (1999) Sequence-divergent units of the ABA-1 polyprotein array of the nematode *Ascaris suum* have similar fatty-acid- and retinol- binding properties but different binding-site environments. Biochemical Journal 340: 337–343. 3. McDermott L, Moore J, Brass A, Price NC, Kelly SM, et al. (2001) Mutagenic and chemical modification of the ABA-1 allergen of the nematode *Ascaris*: Consequences for structure and lipid binding properties. Biochemistry 40: 9918–9926. 4. Kennedy MW (2000) The nematode polyprotein allergens/antigens. Parasitology Today 16: 373–380. 5. Kennedy MW, Britton C, Price NC, Kelly SM, Cooper A (1995) The DVA-1 Polyprotein of the Parasitic Nematode *Dictyocaulus viviparus* - a Small Helix-Rich Lipid-Binding Protein. Journal of Biological Chemistry 270: 19277–19281. 6. Kennedy MW, Allen JE, Wright AS, McCruden AB, Cooper A (1995) The Gp15/400 Polyprotein Antigen of *Brugia malayi* Binds Fatty- Acids and Retinoids. Molecular and Biochemical Parasitology 71: 41–50.
(0.42 MB TIF)
