# Supplementary material for: Solution Structure of a Repeated Unit of the ABA-1 Nematode Polyprotein Allergen of Ascaris Reveals a Novel Fold and Two Discrete Lipid-Binding Sites
Source: PLoS Negl Trop Dis. 2011 Apr 19;5(4):e1040. doi: 10.1371/journal.pntd.0001040 (PMC3079579; doi:10.1371/journal.pntd.0001040)
Supplement: Table S1 — Interhelical angles (in degrees) calculated using QHELIX [1] (0.04 MB DOC) [file pntd.0001040.s001.doc]

**Table S1. Interhelical angles (in degrees) calculated using QHELIX1**

|  | Helix A | Helix B | Helix C | Helix D | Helix E | Helix F | Helix G' |
| --- | --- | --- | --- | --- | --- | --- | --- |
| Helix A |  |  |  |  |  |  |  |
| Helix B | -156.7613 |  |  |  |  |  |  |
| Helix C | -34.3682 | 163.9166 |  |  |  |  |  |
| Helix D | 124.002 | -32.8402 | 150.7511 |  |  |  |  |
| Helix E | -39.3945 | 151.453 | -14.6578 | 139.3209 |  |  |  |
| Helix F | 141.9278 | -21.2213 | 174.7921 | -30.6801 | -169.3773 |  |  |
| Helix G' | -71.5692 | 131.1434 | -38.6145 | -159.5123 | 43.3863 | -143.0433 |  |
| Helix G'' | -12.2954 | -163.0608 | 23.2485 | -131.3819 | 27.1116 | -153.6633 | 61.6219 |

See ref. [1]

1. Lee HS, Choi J, Yoon S (2007) QHELIX: A computational tool for the improved measurement of inter-helical angles in proteins. Protein Journal 26: 556-561.
