# Supplementary material for: Solution Structure of a Repeated Unit of the ABA-1 Nematode Polyprotein Allergen of Ascaris Reveals a Novel Fold and Two Discrete Lipid-Binding Sites
Source: PLoS Negl Trop Dis. 2011 Apr 19;5(4):e1040. doi: 10.1371/journal.pntd.0001040 (PMC3079579; doi:10.1371/journal.pntd.0001040)
Supplement: Table S2 — Helix quality calculated using QHELIX [1] (0.03 MB DOC) [file pntd.0001040.s002.doc]

**Table S2. Helix quality calculated using QHELIX[1]**

|  | residues | h_ir_ (A)^a^ |
| --- | --- | --- |
| Helix A | 5-9 | 0 |
| Helix B | 18-29 | 0.117 |
| Helix C | 34-47 | 0.2186 |
| Helix D | 50-72 | 0.6326 |
| Helix E | 74-86 | 0.1471 |
| Helix F | 90-103 | 0.2821 |
| Helix G' | 106-117 | 0.1896 |
| Helix G'' | 117-124 | 0.1464 |

(a) - average perpendicular distance between the reference data points and the fitted axis

See ref. [1]

1. Lee HS, Choi J, Yoon S (2007) QHELIX: A computational tool for the improved measurement of inter-helical angles in proteins. Protein Journal 26: 556-561.
