# Supplementary material for: Solution Structure of a Repeated Unit of the ABA-1 Nematode Polyprotein Allergen of Ascaris Reveals a Novel Fold and Two Discrete Lipid-Binding Sites
Source: PLoS Negl Trop Dis. 2011 Apr 19;5(4):e1040. doi: 10.1371/journal.pntd.0001040 (PMC3079579; doi:10.1371/journal.pntd.0001040)
Supplement: Table S3 — The Nematode Polyprotein Allergen (NPA) units used to create the multiple alignments in Figures S3 and S4. (0.03 MB DOC) [file pntd.0001040.s003.doc]

**Table S3. The Nematode Polyprotein Allergen (NPA) units used to create the multiple alignments in Figures S3 and S4.**

**Species NPA unit abbreviation UniProtKB Accession code**

*Ascaris suum* As-NPA-1A and -B Q06811

*Dictyocaulus viviparus* Dv-NPA-1A to -L Q24702

*Caenorhabditis elegans* Ce-NPA-1 Q7KNM6

*Loa loa* Ll-NPA-1 Q25303

*Brugia malayi* Bp-NPA-1 A8QEB0

*Wuchereria bancrofti* Wb-NPA-1 Q9GT18

*Ostertagia ostertagi* Oo-NPA-1 Q25594

*Dirofilaria immitis* Di-NPA-1 Q23952

*Onchocerca cervicalis* Oc-NPA-1 O96343

*Acanthocheilonema viteae* Av-NPA-1 O96345

*Toxocara canis* Tc-NPA-1 P91811

*Setaria cervi* Sc-NPA-1 O96344

*Ascaridia galli*  Ag-NPA-1 O61729

*Litomosoides carinii* Lc-NPA-1 O96342

*Heterodera glycines* Hg-NPA-1 Q8TA39
