# Supplementary figures and images for: Solution Structure of a Repeated Unit of the ABA-1 Nematode Polyprotein Allergen of Ascaris Reveals a Novel Fold and Two Discrete Lipid-Binding Sites
Source: PLoS Negl Trop Dis. 2011 Apr 19;5(4):e1040. doi: 10.1371/journal.pntd.0001040 (PMC3079579; doi:10.1371/journal.pntd.0001040)

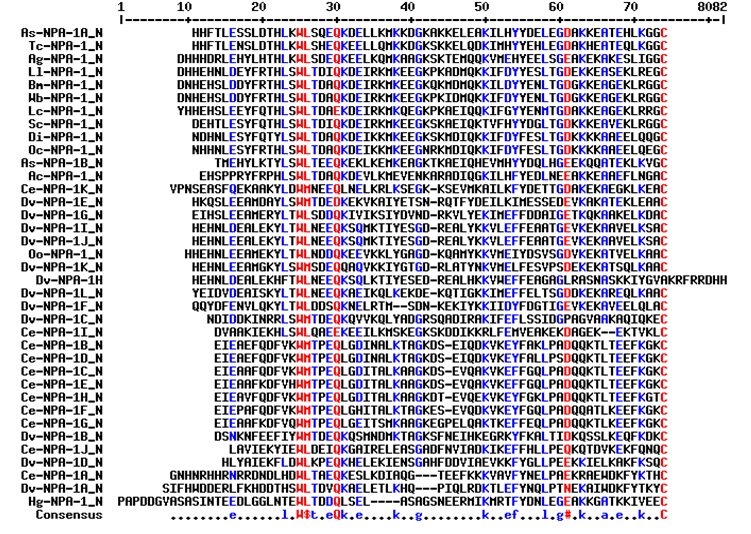

Supplement: Figure S3 — Multiple alignment of the N-terminal halves of NPA units. The sequences are from thirteen species of animal and human parasites, one plant parasite and one free-living species. The sequences of these units are highly divergent and alignments are more informative if created with the N-terminal halves (this Figure; unit names with suffix ‘_N’), ending immediately after the first Cys in the sequences, and C-terminal halves (Figure S4; unit names with suffix ‘_C’) treated separately - see Figure 6 of the main paper for the structural indications that modern day NPA units derive from an ancient duplication event as originally postulated from the ABA-1A sequence [1]. All of the units in the NPA of the cattle parasite Dictyocaulus viviparus and the free-living Caenorhabditis elegans are included, both of which comprise units with highly divergent amino acid sequences. Only partial information on the arrays is available for most parasite species, and sequences of one unit from each is included, except for the two divergent units known from Ascaris suum, in which the units are otherwise almost identical [2]. The units are labeled according to the standard nomenclature for nematode genes and proteins, such that ABA-1A is here labeled As-NPA-1A. The alignment emphasizes the complete conservation of the position of Trp15 in the ABA-1A structure (and position 25 in the alignment), including the adjacent Leu or Met, even in the unusual truncated Dv-NPA-1H unit of D. viviparus. The two cysteines are also absolutely conserved (this Figure and Figure S4), with, again, the exception of the truncated repeat in D. viviparous. The only equivalently conserved position is Gln at position 20 in the structure (and 30 in the alignment), which is replaced just once, with a Glu, a change that can be achieved with a single DNA codon base change. Site-directed substitutions at these positions have various disruptive effects on the thermal stability or ligand binding of ABA-1A [3]. No other posit [file pntd.0001040.s006.tif]

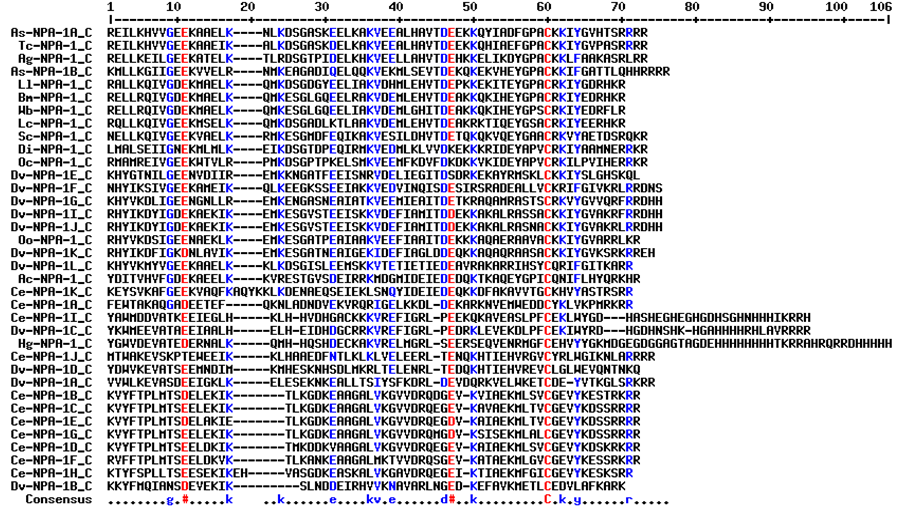

Supplement: Figure S4 — A multiple alignment of amino acid sequences of C-terminal halves of NPA units. The sequences are from thirteen species of animal and human parasites, one plant parasite and one free-living species. The sequences of these units are highly divergent and alignments are more informative if created with the N-terminal halves (Figure S3; unit names with suffix ‘_N’), ending immediately after the first Cys in the sequences, and C-terminal halves (this Figure; unit names with suffix ‘_C’) treated separately - see Figure 6 of the main paper for the structural indications that modern day NPA units derive from an ancient duplication event as originally postulated from the ABA-1A sequence [1]. All of the units in the NPA of the cattle parasite Dictyocaulus viviparus and the free-living Caenorhabditis elegans are included, both of which comprise units with highly divergent amino acid sequences. Only partial information on the arrays is available for most parasite species, and sequences of one unit from each is included, except for the two divergent units known from Ascaris suum, in which the units are otherwise almost identical [2]. The units are labeled according to the standard nomenclature for nematode genes and proteins, such that ABA-1A is here labeled As-NPA-1A. The alignment emphasizes the complete conservation of the position of Trp15 in the ABA-1A structure (and position 25 in the alignment), including the adjacent Leu or Met, even in the unusual truncated Dv-NPA-1H unit of D. viviparus. The two cysteines are also absolutely conserved (this Figure and Figure S3), with, again, the exception of the truncated repeat in D. viviparous. The only equivalently conserved position is Gln at position 20 in the structure (and 30 in the alignment), which is replaced just once, with a Glu, a change that can be achieved with a single DNA codon base change. Site-directed substitutions at these positions have various disruptive effects on the thermal stability or ligand binding of ABA- [file pntd.0001040.s007.tif]
